# Patch-Type Microwave Resonant Sensor Based on a Complementary Split-Ring Resonator for Monitoring Glucose Concentration Under Static and Dynamic Conditions

**DOI:** 10.3390/s26092710

**Published:** 2026-04-27

**Authors:** Wei-Lung Wu

**Affiliations:** Department of Aviation Communication and Electronics, Air Force Institute of Technology, Kaohsiung 820, Taiwan; u0415905@first.nkust.edu.tw

**Keywords:** complementary split-ring resonator, microwave resonant sensor, glucose concentration sensing, noninvasive glucose monitoring, dielectric loading, microstrip sensor, simulated vascular flow platform

## Abstract

**Highlights:**

**What is the main finding of this study?**

- The proposed CSRR-based microwave resonant sensor can distinguish glucose solutions with different concentrations under both static and dynamic conditions by detecting resonance differences. This study indicates the feasibility of integrating electromagnetic sensing and flow-related analysis into a noninvasive monitoring framework.

**Abstract:**

This study designs a complementary split-ring resonator (CSRR)-based 5 GHz patch-type microwave resonant sensor for measuring the concentrations of glucose solutions under static and dynamic conditions. Circulating glucose solutions were used to simulate blood glucose, and the CSRR sensor was operated over a frequency range of 4.8–5.0 GHz. The planar microstrip configuration of the CSRR creates a highly confined electric field within the sensing area. When glucose solution covers or flows through the sensing region, the dielectric loading changes, altering the resonance condition and inducing perturbations. Identifiable measurement features can be extracted from data on the scattering parameter *S*_11_. Glucose solutions with concentrations ranging from 5% to 65% were used to examine the response of the proposed sensor. The concentrations of these solutions were estimated on the basis of resonant frequency shifts, and variation in *S*_21_ at the CSRR’s resonant frequency (or at a fixed frequency corresponding to the maximum slope) was also analyzed.

## 1. Introduction

In the early 20th century, scientists began to explore the development and application of high-frequency electromagnetic waves and microwave resonators that are designed on the basis of the principles of metallic waveguides and resonant cavities. These early devices were typically costly and large. Advancements in materials, fabrication techniques, and solid-state physics led to the emergence of solid-state microwave resonators, whose resonant structures are often made of semiconductor materials [1], such as gallium arsenide [2]. These materials have improved the high-frequency characteristics and efficiency and reduced the size, power consumption, and cost of microwave resonators [3]. Consequently, substantial improvements have been achieved in key technical metrics of microwave resonators, including spectral purity, frequency switching speed, and output frequency range. Over time, microwave resonators have evolved into highly advanced components and are widely deployed across commercial and military systems.

Microwave resonators have various applications in daily life. For example, noninvasive blood glucose monitoring approaches based on microwave resonator technology have been developed to reduce discomfort in patients, particularly those with diabetes [4]. These approaches do not require direct contact with blood or skin; instead, microwave resonators generate electromagnetic waves at frequencies that can penetrate the skin and interact with glucose molecules within the body. Changes in these waves following their interaction with internal molecules—for example, variations in reflected, refracted, or scattered signals—are then analyzed to indirectly quantify blood glucose concentration. The advantages of the aforementioned approaches include painless operation, convenience, continuous measurement ability, and low cost [5]. Furthermore, such approaches can be integrated into smart health-care frameworks, such as those incorporating the Internet of Things, cloud computing, big data analytics, and machine learning, to enable remote monitoring, prediction, and personalized treatment. However, the approaches have limitations related to accuracy, sensitivity, stability, calibration, environmental interference, and interindividual variability.

**Figure 1:**
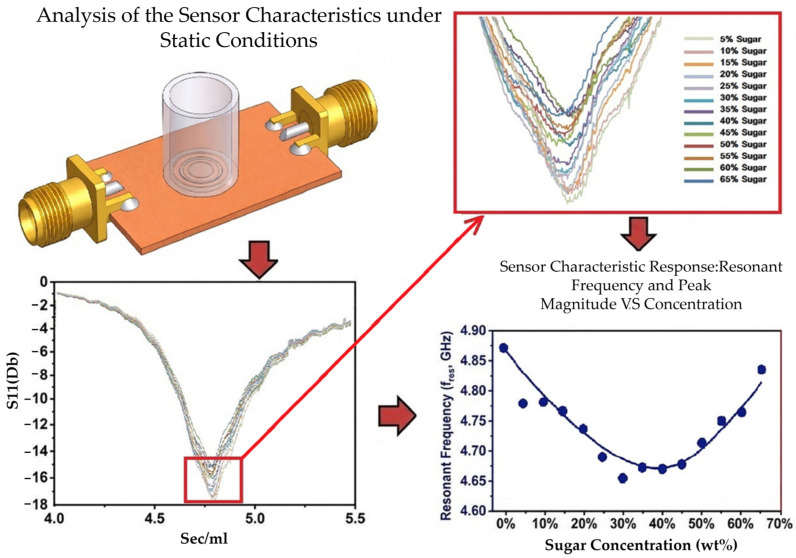
Sensor characterization under design conditions. The figure depicts the *S*_11_ response of the CSRR-based sensor for glucose solution concentrations ranging from 5 to 65 wt% under flow conditions. The enlarged view shows how the response changes with concentration. The resonant frequency increases with increasing glucose concentration, indicating that the sensor can effectively distinguish concentration changes under flow conditions.

**Figure 2:**
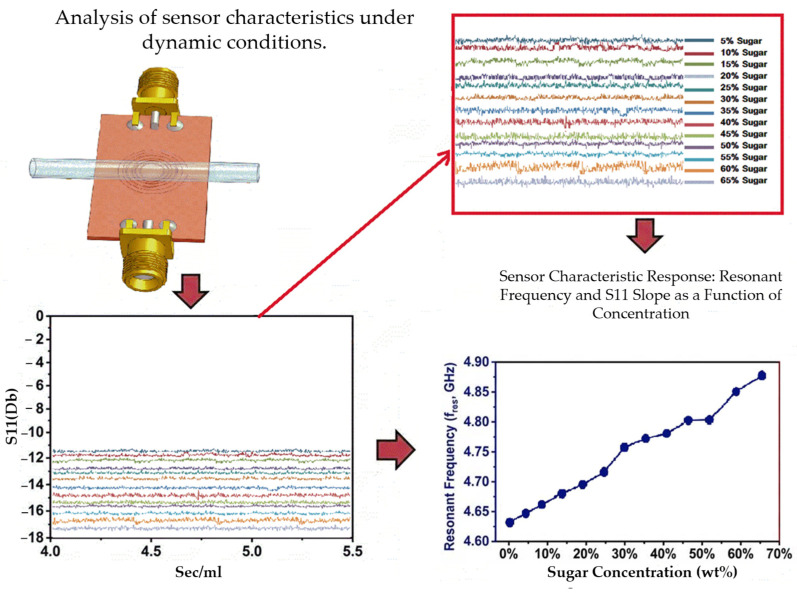
Analysis of sensor characteristics under dynamic conditions. The figure depicts the *S*_11_ response of the CSRR-based sensor for glucose solution concentrations ranging from 5 to 65 wt%. The response was recorded over a 180 s measurement period to evaluate its stability. An enlarged view of the response region and the extracted resonant frequency as a function of glucose concentration are also presented.

This study developed a patch-type microwave resonant sensor based on a complementary split-ring resonator (CSRR). The sensor can measure the concentrations of glucose solutions under both static and design conditions Figure 1 and Figure 2.

The rest of this paper is organized as follows. Section 2 describes the fundamental principles and applications of microwave resonators. Section 3 introduces complementary split-ring resonators (CSRRs), a specific class of microwave resonators, and explains how they can be used to measure the dielectric properties of liquids. It also describes the CSRR-based system developed in this study for measuring glucose concentration. Section 4 presents the experimental results obtained using the proposed system. Finally, Section 5 presents the conclusions and recommendations for future research.

## 2. Operating Principles of Microwave Resonators

A microwave resonator is an electronic component that operates within the microwave frequency band and generates and selects signals at specific frequencies. Microwave resonators play key roles in communications, radar, navigation, and various electronic systems. Microwave resonators operate primarily on the basis of the principle of resonance. The resonance behavior of a microwave resonator is strongly influenced by the structure, dimensional ratios, and material properties of the resonator. At its resonant frequency, a resonator can efficiently store and release electromagnetic energy, thereby selecting signals of a desired frequency. Thus, microwave resonators play an indispensable role in various electronic and communication systems by enhancing frequency selectivity and system stability.

An LC resonator comprises inductors and capacitors connected in series or parallel. Its operating principle is similar to that of a voltage-controlled oscillator. At a specific resonant frequency, the total circuit impedance reaches its maximum or minimum; thus, signals at this frequency are suppressed or transmitted, respectively. As the operating frequency of an LC resonator increases, the quality factor (Q-factor) of the circuit decreases substantially, and the resonator’s frequency-selective performance deteriorates. A microstrip-line resonator consists of a microstrip transmission line of a specific length and is typically implemented on a printed circuit board. When a signal propagates along the microstrip line, a portion of the energy is reflected and interferes with the incident wave, resulting in the formation of a stationary wave. Resonance occurs at frequencies determined by the relationship between the effective electrical length of the transmission line and integer or fractional multiples of the signal wavelength. Microstrip-line resonators are characterized by several key parameters, such as impedance, effective dielectric constant, Q-factor, resonant frequency, and coupling coefficient. The characteristic impedance of a resonator, $Z_{0}$, is the ratio of voltage to current for a wave traveling along the transmission line. For microstrip lines, $Z_{0}$ depends on the geometric properties of the line (e.g., its width *w*), the thickness of the substrate (*h*), and the material properties of the substrate, particularly its relative dielectric constant ($\varepsilon_{r}$). Accurate calculation and design of a resonator’s characteristic impedance are fundamental to achieving impedance matching. $Z_{0}$ serves as a key parameter when determining the appropriate microstrip width and substrate thickness. When w/h≤1, $Z_{0}$ can be expressed as follows:

(1) $$Z_{0}=\frac{60}{\sqrt{\varepsilon_{r}}}\ln\left(\frac{8h}{w}+\frac{w}{4h}\right)$$

Alternatively, when w/h>1, $Z_{0}$ can be expressed as follows:

(2) $$Z_{0}=\frac{120\pi }{\sqrt{\varepsilon_{r}}\left(\frac{w}{h}+1.393+0.667\ln\left(\frac{w}{h}+1.444\right)\right)}$$

where w, h, and $\varepsilon_{r}$ represent the width of the microstrip line, the thickness of the substrate, and the relative dielectric constant of the substrate, respectively. Equations (1) and (2) are derived by solving the electromagnetic wave equations under the relevant boundary conditions while accounting for the mixed-field effects of the electric field in the dielectric substrate and air, thereby yielding the electric and magnetic field distributions of the microstrip line. For an open-circuited microstrip-line resonator, the physical length *L* is approximately one quarter of the wavelength: L=λ/4. The relationship between the wavelength λ, propagation velocity v, and frequency f is λ=v/f. The following expression is obtained by substituting this relationship into L=λ/4 and rearranging the terms: f=v/4L. The physical parameters of the microstrip line, such as its length, width, substrate thickness, and dielectric constant, are key factors influencing $f_{r}$, as shown in the following equation: Equation (3)

(3) $$f_{r}=\frac{v}{2L}=\frac{c}{2L\sqrt{\varepsilon_{eff}}}$$

Although the formulas for calculating the resonant frequency $f_{r}$ for the ElectricField and Magnetic Field modes are similar in form, their mode number requirements and boundary conditions differ, resulting in distinct $f_{r}$ values and field distributions. For a cavity resonator, the total Q-factor is expressed as $Q_{total}=1/Q_{c}+1/Q_{d}+1/Q_{r}$, where $Q_{c}$, $Q_{d}$, and $Q_{r}$ are the Q-factors associated with the metallic conductor, dielectric material, and radiation loss, respectively. The impedance-matching performance of a cavity resonator is primarily influenced by its characteristic impedance, which is typically controlled by the geometry of its cavity. The dielectric properties of the material inside the cavity affect the effective dielectric constant $\varepsilon_{eff}$ and thereby influence the resonant frequency.

## 3. CSRRs

**Figure 3:**
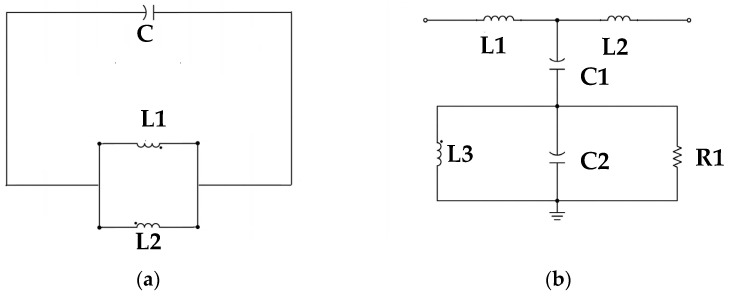
(**a**) Structure of a CSRR, including coupling capacitance C_1_ and symmetric inductive elements L_1_ and L_2_. (**b**) Equivalent circuit model of the CSRR.

CSRRs are resonant structures derived from split-ring resonators with a complementary geometry. A CSRR consists of two concentric split rings etched in a complementary (slot-type) configuration, with their openings positioned opposite each other. The structure is typically etched on the ground plane, and the conductive layer of a microstrip line or coplanar waveguide contain slot apertures, enabling the resonator to exhibit a tuned electromagnetic response. CSRRs are planar printed resonators (microstrip or slot-line configuration), but their resonance mechanism can be accurately described using a lumped-element equivalent LC circuit model. Therefore, CSRRs combine the characteristics of microstrip structures and LC-based resonators. Figure 3a illustrates the structure of the CSRR-based sensor.

**Figure 4:**
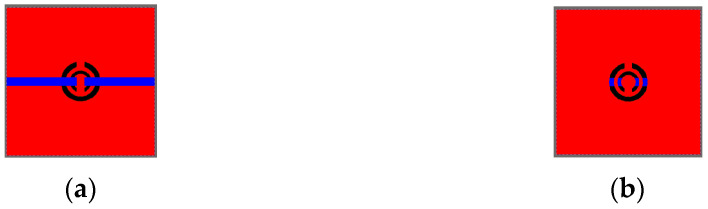
(**a**) Simplified equivalent circuit of a CSRR (**b**) PCB backside.

An equivalent inductance is generated when the two etched concentric ring slots of a CSRR are excited by a magnetic field. These slots function as two series inductors (each labeled L/2) and influence the CSRR’s resonant frequency. The capacitance $C_{c}$ represents the electric field coupling across the narrow gap between the slots. This capacitance is partly responsible for the resonant behavior of the CSRR and strongly affects its resonance characteristics. The parallel inductance $L_{r}$, located in the lower left of the equivalent circuit in Figure 3b, represents parasitic inductance from slot edges or other structural regions beyond the primary ring path, which affects CSRR performance at high frequencies. The parallel capacitance $C_{r}$ represents parasitic capacitance introduced by the substrate and its environment. In the equivalent circuit model, $C_{r}$ captures the effect of parasitic capacitance on the frequency response. The parallel resistor *R* represents energy dissipation due to metallic and dielectric losses. It can model intrinsic energy loss in the CSRR structure and reduce the resonator’s Q-factor. These circuit elements represent the physical characteristics of a CSRR, including primary resonance, loss mechanisms, and parasitic effects. Figure 4a,b presents the simplified equivalent circuit of a CSRR.

The annular slots of a CSRR form a closed loop. When electromagnetic waves propagate through the CSRR structure, currents are induced within the slots, and these currents generating magnetic fields that produce an equivalent inductance, which is expressed as $k^{\prime }\;=\sqrt{1-k^{2}}$, where $k=\frac{W}{W+2G}$. which is expressed by Equation (4). leads to a simplified equivalent LC circuit model. for which the resonant frequency [Equation (4)] can be calculated if $C_{c}$ and $L_{0}$ are known. The capacitance $C_{c}$ is expressed in Equation (5), and $L_{0}$ is calculated using Equation (6).

(4) $$f_{r}=\frac{c}{2\pi a\sqrt{\varepsilon_{r}}}\sqrt{{\left(\frac{x_{mn}}{a}\right)}^{2}+{\left(\frac{m\pi }{h}\right)}^{2}}$$

(5) $$C_{c}=\frac{\pi^{3}\varepsilon_{0}}{c^{2}}\int_{0}^{\infty }\frac{{\left[bB\left(kb\right)-aB\left(ka\right)\right]}^{2}}{k^{2}}\left[0.5\left(1+\frac{1+\frac{\varepsilon }{\varepsilon_{0}}\tanh\left(kh\right)}{1+\frac{\varepsilon_{0}}{\varepsilon }\tanh\left(kh\right)}\right)\right]dk$$

(6) $$L_{0}=\frac{\pi^{3}\mu_{0}}{4c^{2}}\int_{0}^{\infty }\frac{{\left[bB\left(kb\right)-aB\left(ka\right)\right]}^{2}}{k^{2}}dk$$

When glucose is introduced into the resonator cavity, the relative permittivity of the material ($\varepsilon_{r}$) alters the resonant frequency of the cavity. On the basis of the change in this frequency, the dielectric constant can be derived using the expression $\varepsilon_{r}={\left(f_{0}/f\right)}^{2}$, where $f_{0}$ and f are the resonant frequencies before material insertion (empty cavity) and after material insertion, respectively. This method can also be used to determine the dielectric loss factor tan δ. The Q-factor after material insertion (Q) and tan δ have the following relationship: Q=1/tan δ. Once f has been determined, the 3 dB bandwidth can be identified to calculate the corresponding Q-factor. The dielectric loss factor can then be obtained from the relationship Q=1/tan δ. Numerous other techniques for measuring dielectric properties are available, such as the transmission line, reflection, free space, resonance, dielectric analyzer, coplanar waveguide, vector network analyzer, and thermal analysis methods and time-domain reflectometry (Table 1).

## 4. Measurement of Glucose Concentration Changes and Long-Term Flow Behavior

### 4.1. Sensor Simulation

**Figure 5:**
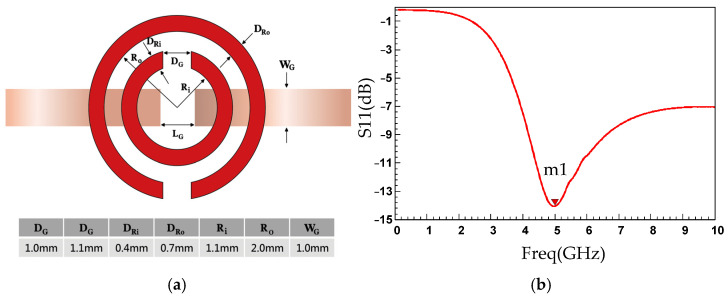
(**a**) Circuit parameters and (**b**) simulated $S_{11}$ values of the designed CSRR.

Glucose solutions of various concentrations were analyzed using a CSRR-based resonant circuit. The resonant frequency of a CSRR varies with the dielectric constant of the medium being analyzed. Because the dielectric constant of a glucose solution is affected by its glucose concentration, the resonant frequency of the CSRR was affected by glucose concentration. This effect enabled the determination of glucose concentration. First, a CSRR was operated at a frequency of 5 GHz. The ANSYS High Frequency Structure Simulator 15.0 (ANSYS Inc., Canonsburg, PA, USA) software was then used to simulate electric field distribution, magnetic field distribution, resonant frequency, and S-parameters of the CSRR. First, a CSRR was operated at a frequency of 5 GHz. The results of this operation were then used in ANSYS High Frequency Structure Simulator 15.0 software to simulate Subsequently, a resonant circuit capable of measuring glucose concentrations was fabricated on the basis of the simulated characteristics. Figure 5 presents the CSRR circuit parameters determined using the ANSYS High Frequency Structure Simulator.

In Figure 5, $D_{G}$ is the width of the gap in each ring, which was identical for the inner and outer rings; $L_{G}$ is the length of the two microstrip feed lines, between which electromagnetic waves were coupled through the CSRR structure; $D_{Ri}$ and $D_{Ro}$ are the slot widths of the inner and outer rings, respectively; $R_{i}$ and $R_{o}$ are the radii of the inner and outer rings, respectively; and $W_{G}$ is the width of the microstrip feed lines.

Figure 5b depicts the simulated $S_{11}$ response of the circuit depicted in Figure 5a. At marker m1, the reflection coefficient was −14.7946 dB, and the frequency was 5 GHz; therefore, the resonant frequency of the CSRR was 5 GHz. Setting the resonant frequency of the CSRR at 5 GHz for noncontact glucose concentration measurement offers several advantages. First, the relatively high frequency means that the system is highly sensitive to minor dielectric variation, enabling precise detection of changes in glucose concentration. Second, electromagnetic waves with a frequency of 5 GHz can penetrate aqueous solutions to a moderate depth, meaning that they can interact sufficiently with the solutions without excessive absorption. In addition, 5 GHz is a frequency band commonly used in wireless communications, and the associated measurement equipment and technologies are well established, ensuring instrument availability and ease of calibration.

### 4.2. Sensor Implementation

**Figure 6:**
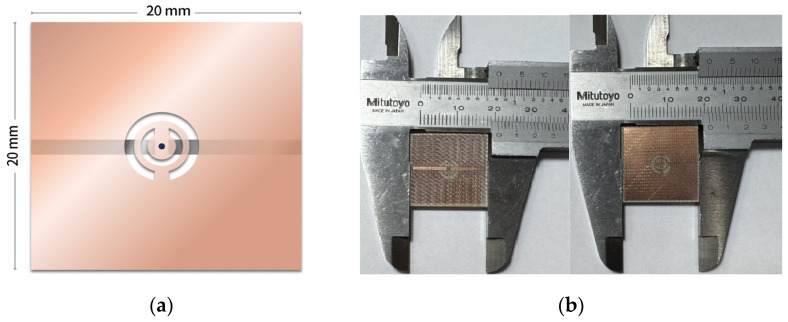
(**a**) Simplified equivalent circuit model of the CSRR and (**b**) front (**left**) and back (**right**) views of the fabricated CSRR circuit.

Figure 6a depicts the simulated model of the CSRR circuit, and Figure 6b presents the front and back views of the fabricated CSRR circuit. The front side consisted of two microstrip feed lines. The circuit was fabricated on a printed circuit board with dimensions of 20 mm × 20 mm by using FR-4 glass epoxy substrate with a thickness of 0.4 mm. The relative permittivity (Hot spot/f_res) $\varepsilon_{r}$ was 4.4, and the loss tangent tan δ was 0.02. The top and bottom surfaces were coated with 35 μm thick copper layers, and the intermediate FR-4 dielectric layer had a thickness of 0.33 mm.

**Figure 7:**
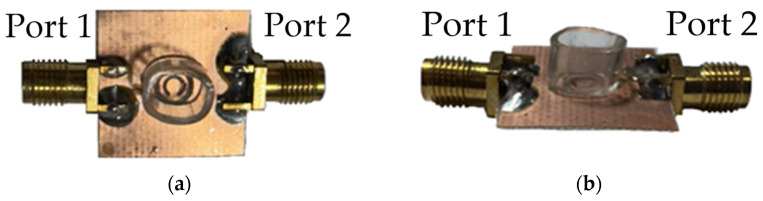
(**a**) Front and (**b**) side views of the fabricated CSRR with an empty liquid container.

**Figure 8:**
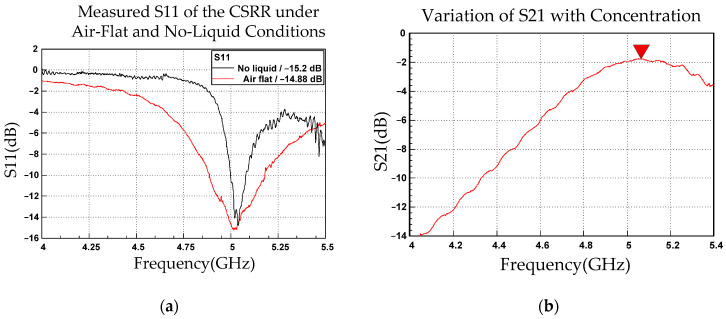
(**a**) $S_{11}$ responses under air-flat and no-liquid conditions. (**b**) The figure presents the S_21_ values measured in the presence of the liquid container, with a labeled point at 5.0656 GHz (−1.832 dB).

Figure 7a and Figure 7b depict the front and side views, respectively, of the fabricated CSRR with a small liquid container. The split-ring structure of the CSRR induced localized resonance, generating strong electric and magnetic fields. These resonant modes enabled electromagnetic coupling from Port 1 to Port 2 through the CSRR structure on the back side. The thickness of the circuit board was 0.4 mm. Subminiature version A connectors were attached to the terminals of Ports 1 and 2 and connected to a vector network analyzer, which measured corresponding S-parameters. This configuration constituted a simple 5-GHz resonant circuit. A small plastic container was fixed above the split-ring region to facilitate the measurement of glucose solutions of various concentrations. This container caused a slight reduction in resonant frequency; therefore, the CSRR’s resonant frequency was fine-tuned to 5.02 GHz. Figure 8a presents the measured $S_{11}$ response of the fabricated resonator. After the attachment of the empty container, the resonant frequency remained approximately 5 GHz but decreased by approximately 2 MHz compared with the condition without the container. This frequency shift is attributed to the dielectric properties of the container material plastic 2.7 permittivity (ε_r_) in this study. The $S_{11}$ value at 5 GHz was −14.815 dB. Signal transmission from Port 1 to Port 2 of the CSRR corresponds to the $S_{21}$ parameter, which reflects the transmission characteristics of a signal as it propagates through the CSRR structure. Therefore, $S_{21}$ was used to evaluate the response of the CSRR to changes in glucose concentration. Figure 8b presents the $S_{21}$ values measured in the presence of the liquid container. The $S_{21}$ value was maximum (−1.832 dB) at a frequency of 5.0656 GHz.

### 4.3. Electromagnetic Field Distribution Analysis

**Figure 9:**
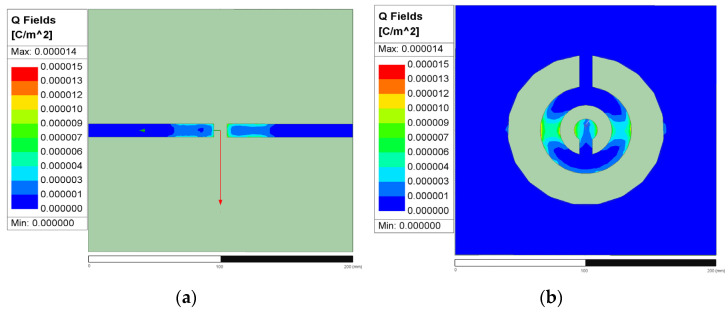
The figure presents the surface charge density (Q-field) distributions (unit: C/m^2^) for the front and back sides, respectively: (**a**) front side and (**b**) back side. The X (red), Y (green), and Z (red) directions are also indicated.

**Figure 10:**
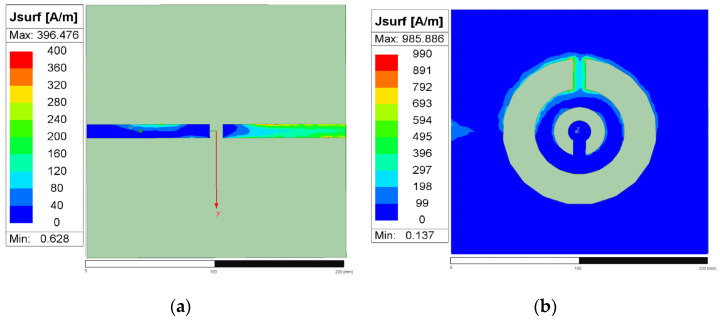
The figure presents the surface current density density *J*_surf_ (unit: A/m) for (**a**) the front side and (**b**) the back side, with the X (red), Y (green), directions indicated.

**Figure 11:**
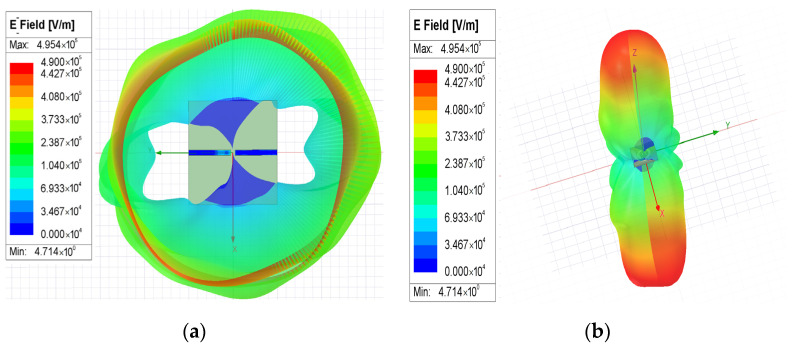
Electric-field (E-field) distribution (unit: V/m): (**a**) front view and (**b**) back view. The X (red), Y (green), and Z (blue) directions are indicated.

The simulated electromagnetic distributions of the proposed CSRR revealed that the resonant structure comprises two conductive sections (located on the located either side of the split in the ring) separated by a narrow central in-ring gap that serves as the principal coupling and sensing region. This geometrical discontinuity is the location at which the electromagnetic energy is most strongly confined and the interaction with the material being tested is the strongest. As shown in the Q-field distributions presented in Figure 9a,b, charge density mainly concentrates around the in-ring gap and the adjacent edges of the left and right conductors. This result indicates surface charge accumulation in the vicinity of the gap, which is a characteristic feature of the capacitive region of a resonant structure. The transition from blue to cyan in Figure 10 corresponds to an increase in surface charge density from lower to higher values. Although the color scale extends to red, the actual distribution is predominantly limited to the blue-to-cyan range, which indicates that the figure mainly reflects an overall trend and does not indicate high charge saturation. Figure 10a,b presents the surface current density (*J*_surf_) distributions, which suggest that the induced surface current is primarily guided along the conductive paths on the two sides of the outer ring and is perturbed in the vicinity of the in-ring gap. This behavior indicates that the left and right conductive sections function as the main current-carrying paths and that the in-ring gap interrupts the current continuity and enhances electromagnetic localization. Accordingly, current redistribution and charge accumulation together strengthen the local resonant response in the gap region. The electric-field distributions presented in Figure 11a,b underscore that the field confinement is strongest around the central gap and neighboring slot edges, where the electric energy is highly localized. This field concentration is consistent with the observed Q-field pattern because greater surface charge accumulation generally corresponds to stronger local electric fields. Therefore, the in-ring gap region can be identified as the dominant sensing zone of the proposed CSRR.

Overall, the simulated Q-field, *J*_surf_, and E-field results consistently indicate that the electromagnetic response is not uniformly distributed across the structure but is mainly concentrated near the gap in the outer ring. This localized behavior is crucial for dielectric sensing because the gap region is the part most sensitive to changes in dielectric constant. When a liquid covers this region, even a small variation in dielectric properties can perturb the local field distribution, modifying the effective capacitance of the resonator and thereby changing its resonance characteristics. This explains why the gap plays the key role in the sensing mechanism of the proposed CSRR-based sensor.

### 4.4. Experimental Results, Static Measurement, and Statistical Analysis

**Figure 12:**
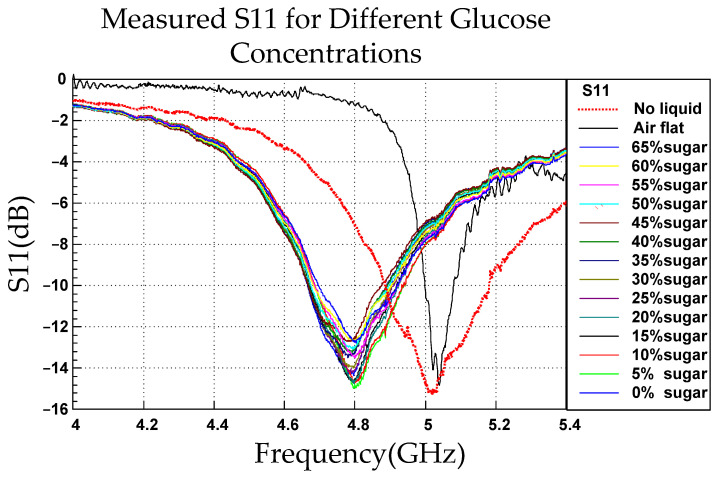
$S_{11}$ Measured S11 values of the CSRR sensor under the air-flat condition in the absence of liquid flow. and no-liquid conditions and for glucose solutions of various concentrations.

In the physical experiment, a reference line was marked on the small container affixed to the CSRR, and glucose solutions with concentrations ranging from 0% to 65% (in increments of 5%; 65% approached the saturation concentration) were then added to the container. For each concentration, precisely enough liquid to fill the container to the marked line was added to ensure a consistent volume. Each concentration was measured five, and corresponding $S_{11}$ values—presented in Figure 12—were averaged.

**Figure 13:**
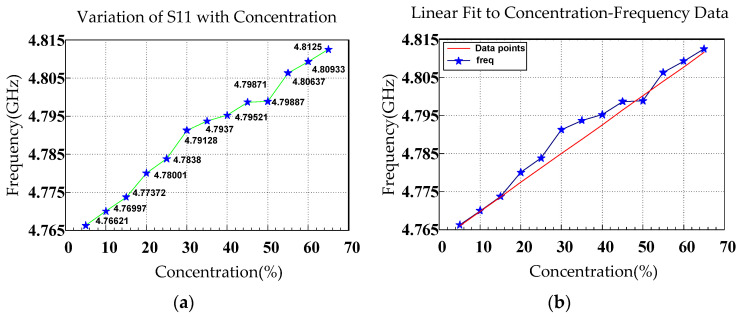
(**a**) Resonant frequency obtained from the $S_{11}$ response of the CSRR at various glucose concentrations. (**b**) Fitted curve of the resonant frequency versus glucose concentration.

Figure 13a,b depicts resonant frequency as a function of glucose concentration. When pure water (0% glucose) was added to the 5-GHz resonator, the resonant frequency was approximately 4.8 GHz. With increasing glucose concentration, the resonant frequency increased in a linear trend. The minimum $S_{11}$ value at each concentration was identified to comprehensively examine the frequency shifts and magnitude variations.

**Figure 14:**
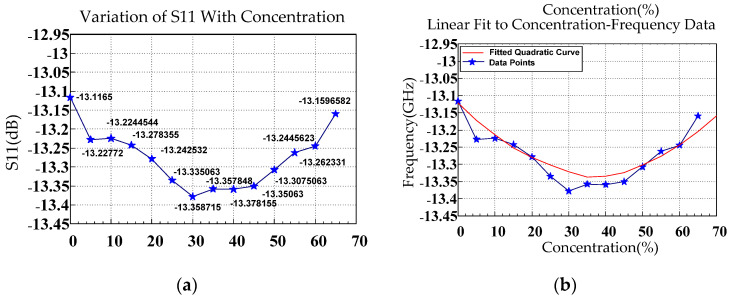
(**a**) *S*_11_ as a function of glucose concentration. (**b**) Fitted curve for the resonant magnitude.

The approximately linear increase in resonant frequency with glucose concentration was attributable to the difference in dielectric constant between water and glucose. The dielectric constant of water is higher than that of glucose. Therefore, with increasing glucose concentration, the effective dielectric constant of the solution decreased, and the velocity at which electromagnetic waves propagated in the medium increased, thereby increasing the resonant frequency. Because the resonant condition of the CSRR structure is influenced by its dielectric environment, a reduction in relative permittivity increases the resonant frequency, reflecting the inverse relationship given by $f\propto 1/\sqrt{\varepsilon_{r}}$. The equation for the linear fit to the curve in Figure 14a, was y=673,626.3736x+4,765,964,285.7143, Figure 14b where y is the frequency and x is the glucose concentration. where y represents the frequency and x represents glucose concentration. The slope m was 673,626.3736 Hz/%, indicating that for every 1% increase in glucose concentration, the resonant frequency increased by approximately 673,626.3736 Hz. The intercept b was 4,765,964,285.7143 Hz, corresponding to a resonant frequency of approximately 4.766 GHz at a glucose concentration of 0%. The fitted linear relationship can predict the resonant frequency at various glucose concentrations and thus guide quantitative analysis.

Unlike the relationship between glucose concentration and the resonant frequency derived from the $S_{11}$ response, that between glucose concentration and the resonant magnitude obtained from the same response was approximately quadratic. This finding may be attributable to the polarization characteristics of the solution and intermolecular interactions that influenced the effective dielectric constant. An increase in glucose concentration altered the dielectric loss characteristics of the aqueous solution. With increasing glucose concentration, the dielectric loss increased, reducing the reflection coefficient. When the concentration exceeded a critical threshold, the loss decreased, increasing the reflection coefficient. A change in glucose concentration altered the distribution of the electric field within the medium, thereby altering the reflection coefficient. These combined effects likely accounted for the nonlinear variation observed in the S-parameters.

At the resonant frequency, the CSRR exhibited its maximum $S_{21}$ value, corresponding to the optimal transmission performance. This result was obtained because at resonance, the CSRR exhibits maximum impedance matching, minimum signal loss, and maximum transmission efficiency. Because the resonant frequency of a CSRR is highly sensitive to the dielectric constant of the medium, the concentration of a glucose solution can be inferred by measuring the frequency at which $S_{21}$ reaches its peak. $S_{11}$ represents the reflection characteristics at the input port, whereas $S_{21}$ represents the overall transmission characteristics of the system. By contrast, $S_{22}$ represents the reflection characteristics at the output port. However, $S_{22}$ does not directly reflect the effect of changes in the dielectric constant on the overall system response. It is relatively insensitive to changes in dielectric properties and requires high-precision measurements at the output port. These characteristics would introduce additional complexity to the experimental setup and data processing. Therefore, in both research and practical applications, mostly $S_{11}$ and $S_{21}$ are used in measurements and analyses.

In the low-concentration range, interactions between water molecules were predominant. The presence of a small quantity of glucose disrupted the hydrogen-bonding network of water, resulting in a substantial reduction in the dielectric constant of the solution. With increasing glucose concentration, the dielectric loss considerably increased, sharply reducing the reflection coefficient. Because the polarization behavior of water molecules governed the dielectric properties of the solutions, even a small quantity of glucose markedly altered the polarization mechanism. In the high-concentration region, interactions between glucose molecules became dominant, and glucose molecules began to form a network-like structure. Under these conditions, intermolecular interactions markedly altered the structural organization and dielectric properties of the solution, and the solution’s dielectric loss increased again. The polarization behavior of glucose molecules began to strongly influence the overall dielectric characteristics, inducing a large change in the dielectric constant. Consequently, the fitting curve for the relationship between glucose concentration and resonance magnitude was quadratic.

**Figure 15:**
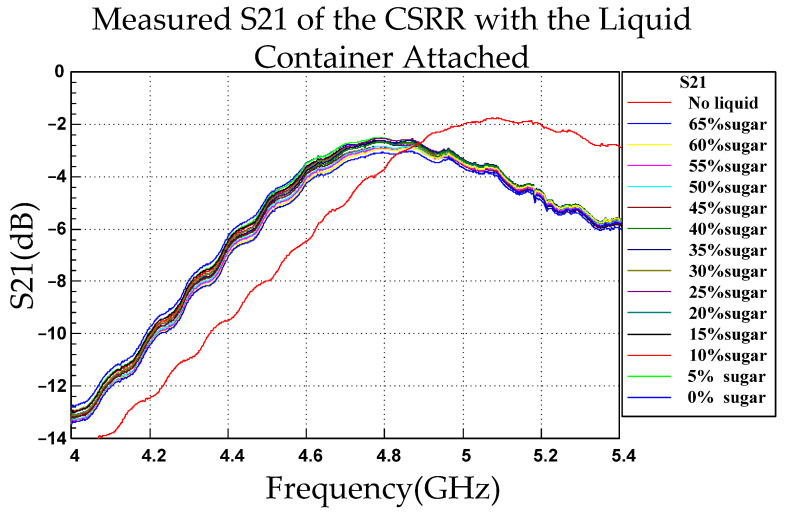
$S_{21}$ values of the CSRR as a function of frequency with the liquid container attached and under various glucose concentrations.

Figure 15 depicts the $S_{21}$ responses for various glucose concentrations. When the glucose concentration was 0% (pure water), the resonant frequency of the CSRR was approximately 4.77 GHz. The resonant frequency increased with increasing glucose concentration. This trend was identical to that indicated by the $S_{11}$ measurements; however, the resonant frequencies obtained from the $S_{11}$ and $S_{21}$ measurements differed slightly because $S_{11}$ and $S_{21}$ represent different physical quantities. $S_{11}$ measures the portion of a signal reflected back to the input port, whereas $S_{21}$ measures the portion transmitted from the input port to the output port.

**Figure 16:**
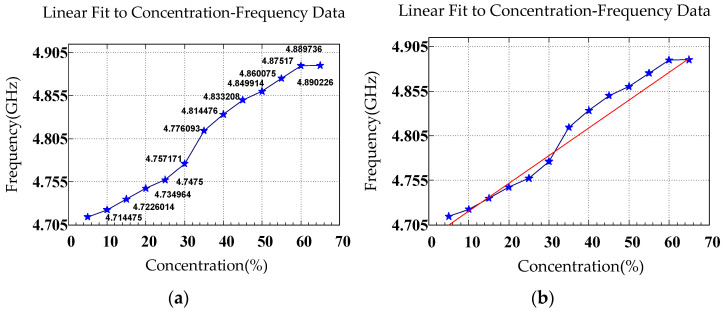
(**a**) Resonant frequency obtained from the $S_{21}$ response of the CSRR at various glucose concentrations. (**b**) Linear fit to the curve in (**a**).

Figure 16a,b depicts resonant frequency as a function of glucose concentration where the data were obtained from the CSRR’s $S_{21}$ response. The relationship between resonant frequency (*y*) and glucose concentration (*x*) was a positive linear trend described by the equation y=1,421,978.02x+4,764,857,140. The slope m was 1,421,978.02 Hz/%, indicating that for every 1% increase in glucose concentration, the resonant frequency increased by approximately 1,421,978.02 Hz. The intercept b was 4,764,857,140 Hz, corresponding to a resonant frequency of approximately 4.765 GHz at a glucose concentration of 0%. Table 2 presents the focuses, resonance characteristics, and findings of $S_{11}$ and $S_{21}$ measurements.

Table 3 lists the mean, resonant frequency, and *S*_11_ magnitude extracted from the measured responses at various glucose concentrations, including the mean (μ), standard deviation (SD; σ), and μ ± nσ ranges. The results indicated that the resonant frequency in Figure 13 and Figure 16 increased with increasing glucose concentration, whereas the *S*_11_ magnitude in Figure 14 exhibited nonmonotonic variation. The relatively small SD values suggest favorable measurement stability and repeatability.

As shown in Table 4, the proposed sensor differs from previous CSRR-based sensors in that it can be used under both static and dynamic conditions and that flow-related measurements are integrated into the sensing framework.

### 4.5. Experimental Results, Dynamic Measurement, and Statistical Analysis

**Figure 17:**
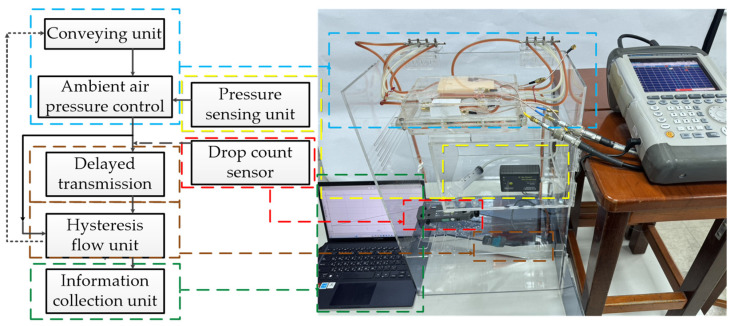
Architecture of the simulated vascular blood flow detection device.

Finally, the proposed simulation platform, named the Simulated Vascular Blood Flow Detection Device, was used to simulate human blood circulation and measure glucose concentrations under controlled conditions. This platform consisted of a conveying unit, an ambient air pressure controller, a pressure-sensing unit, a Volume per Drop sensor, a delayed transmission unit, a hysteresis flow unit, and an information collection unit (Figure 17) [14].

Drop count and pressure were synchronously measured. Flow-related indicators under various glucose concentrations were recorded to simulate human blood flow behavior.

In subsequent simulations, the device parameters were adjusted to reflect the physiological characteristics of human blood flow. The measurement modules of the proposed system are presented in Table 5.

**Figure 18:**
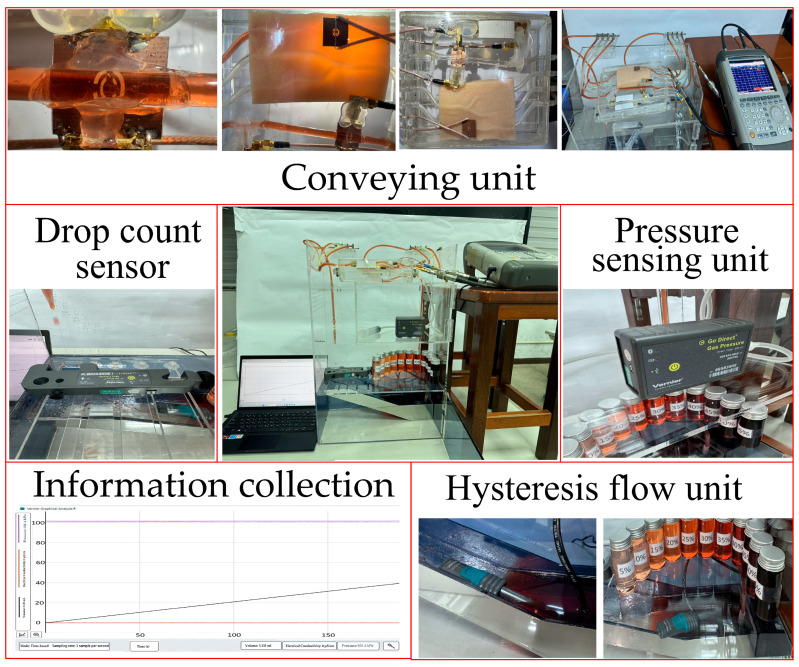
Components and functional layout of the Simulated Vascular Blood Flow Detection Device.

The Simulated Vascular Blood Flow Detection Device was developed to provide a reproducible benchtop platform for analyzing liquid behavior under controlled, physiologically relevant conditions. Figure 18 depicts the components and functional layout of the device. The system is organized into five functionally coupled modules: a conveying unit, a drop-count sensing unit, a pressure sensor, a pneumatic control unit, a hysteresis flow unit and information collection module, and a processing unit. Together, these modules establish stable flow boundary conditions, acquire flow-related signals synchronously, and generate quantitative indicators (drop rate, flow rate, and pressure metrics) for comparative analyses of various glucose concentrations.

**Figure 19:**
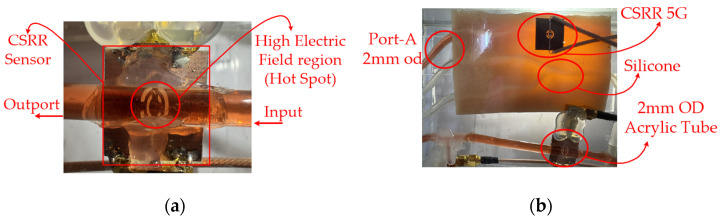
(**a**) CSRR sensor and high electric-field sensing region. (**b**) Experimental setup with acrylic tube (2 mm OD) and silicone interface for liquid measurement.

**Figure 20:**
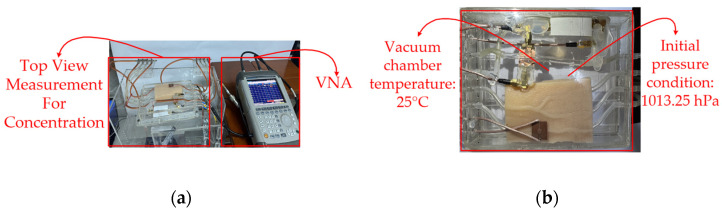
(**a**) Top-view of the concentration measurement setup with VNA. (**b**) Vacuum chamber environment at 25 °C and 1013.25 hPa.

**Figure 21:**
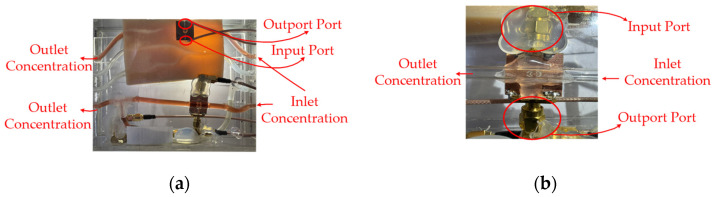
(**a**) Flow-based concentration measurement setup with CSRR sensor. (**b**) Side view showing inlet/outlet concentration and port configuration.

Conveying unit: The conveying unit reproducibly delivers glucose solution to the flow channel, providing stable conditions for both drop-based and continuous-flow measurements. A fixed drop volume of 1 mL and a measurement duration of 180 s were used to standardize the experiments, and measurements were conducted at the same time and location. Although the conveying unit did not directly generate conductivity-related signals, it established the flow boundary conditions, including the delivery pressure, flow channel impedance, and liquid viscous resistance, which determined the drop rate and pressure response. Figure 19 depicts (a) the top view of the CSRR-based sensor integrated with the fluidic channel. The high electric field region (hot spot) is concentrated at the CSRR structure, where strong interaction with the flowing liquid occurs. (b) Side view of the CSRR sensing structure, showing the microstrip configuration, SMA port connections (2 mm OD), and the acrylic flow channel sealed with silicone. Figure 20 depicts the (a) top-view measurement setup for concentration analysis, showing the integration of the CSRR sensing module with the vector network analyzer (VNA) for electromagnetic characterization. (b) Experimental chamber under controlled conditions, indicating an initial pressure of 1013.25 hPa and a temperature of 25 °C. Figure 21 depicts the (a) side view of the fluidic sensing setup, illustrating the inlet and outlet concentrations of the liquid flowing through the channel above the CSRR sensor. (b) Top view of the sensing region, showing the input and output ports connected to the CSRR structure, along with the corresponding inlet and outlet concentration paths.

**Figure 22:**
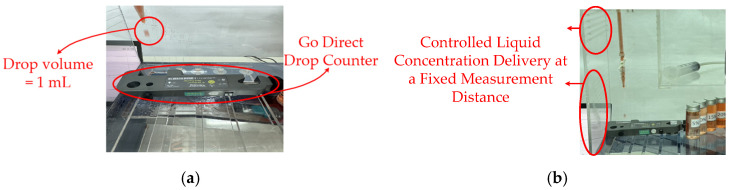
(**a**) Photograph of the Go Direct drop counter used to quantify droplet volume in the flow measurement system. (**b**) Controlled liquid delivery setup for concentration-dependent measurements at a fixed sensing distance.

Drop count sensor The drop count sensor converted fluid flow into a drop count by using optical, infrared, or electrical sensing mechanisms to detect individual drops. Each detected drop generated a pulse signal, and these signals were used to calculate the drop rate. With increasing glucose concentration, the drop count decreased, indicating that a lower drop rate was associated with higher viscosity and flow resistance. The flow rate statistics obtained from the experiment are summarized in Table 6. Figure 22 depicts the (a) drop counting module using a Go Direct drop counter, where each droplet corresponds to a fixed volume of 1 mL. (b) Experimental setup for controlled liquid concentration delivery at a fixed measurement distance.

**Figure 23:**
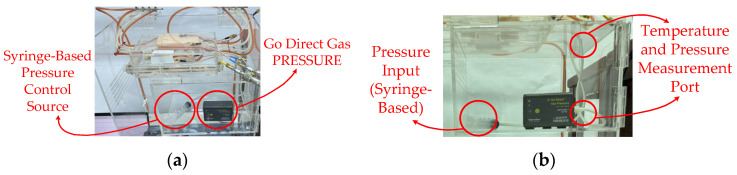
(**a**) Syringe-based pressure control source and Go Direct gas pressure sensor. (**b**) Pressure input and temperature/pressure measurement port.

Pressure-sensing unit pressure was measured to evaluate flow impedance. It served as both the force driving the flow and an indicator of flow resistance. Under fixed driving conditions, a higher glucose concentration led to greater flow resistance, as reflected by a lower drop rate, higher pressure demand, and an altered pressure fluctuation pattern. The following pressure-related variables were monitored: mean pressure, pressure fluctuation (SD), and time taken to transition from the initial transient state to a steady state. Air pressure was controlled to adjust the driving pressure differential. Figure 23 depicts the (a) pressure control module using a syringe-based pressure source and a gas pressure sensor for real-time monitoring. (b) Pressure input and measurement configuration, showing the syringe-based pressure input and the temperature and pressure measurement port.

**Figure 24:**
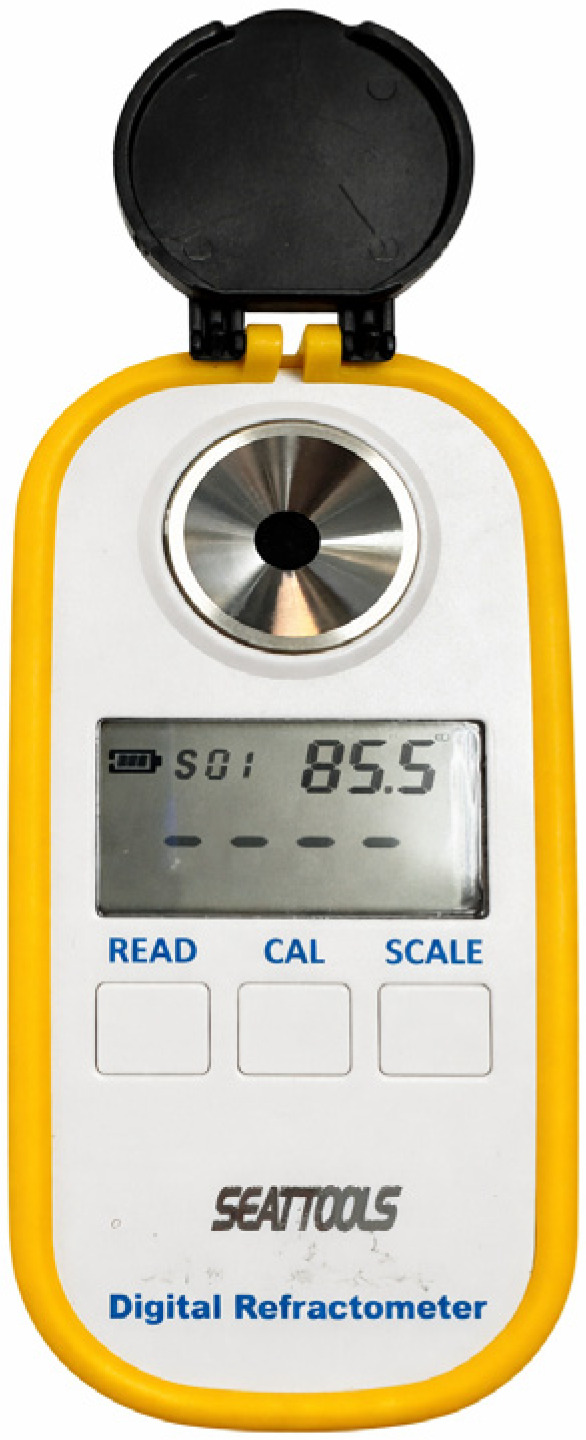
Digital refractometer employed to measure the refractive index of the glucose solutions, providing reference values for concentration-dependent dielectric analysis.

Information collection unit: The information collection unit recorded pressure waveforms and acquired pulse signals from the drop count sensor to calculate the drop frequency (drops per second). It synchronized the signals in time, calculated the flow rate, and generated reports and tables. All measurements were integrated over a fixed duration of 180 s. On the basis of the measured drop frequency and the fixed single-drop volume of 1 mL, the flow rate (in milliliters per minute) and volume delivered over 3 min (in milliliters per 3 min) were calculated. Pressure signals were also recorded as an auxiliary indicator of flow impedance. Finally, the unit integrated the drop rate and pressure data to analyze blood flow behavior and generate results. A digital refractometer (Digital refractometer MET-PSM+2 (0–90% Brix), The glucose concentration (Brix value) was measured using a digital refractometer (SEATOOLS, Brooklyn, NY, USA) was used to obtain the ground-truth measurements of glucose concentrations, shown in Figure 24.

**Figure 25:**
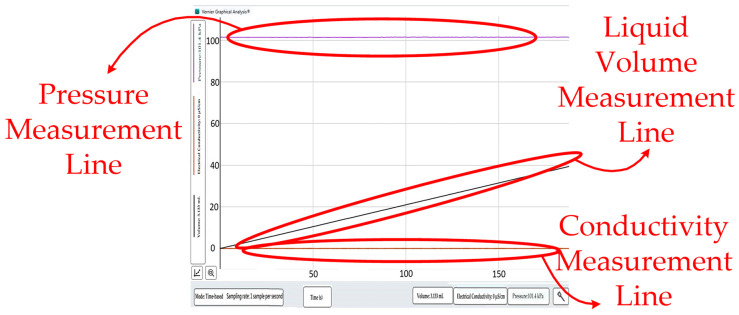
Time-domain signals of pressure, liquid volume, and conductivity measurements.

Conversion and comparison notes: The refractometer yielded measurements in terms of Brix%. For dilute aqueous solutions, a simple conversion was used to estimate the glucose-equivalent concentration from Brix%. One degree Brix (°Bx) corresponds to approximately 1.01 g of glucose in 100 g of solution, that is, a mass fraction of 1%. The calculation is expressed as follows. Figure 25 depicts multi-parameter measurement results showing the pressure measurement line, liquid volume measurement line, and conductivity measurement line.

**Figure 26:**
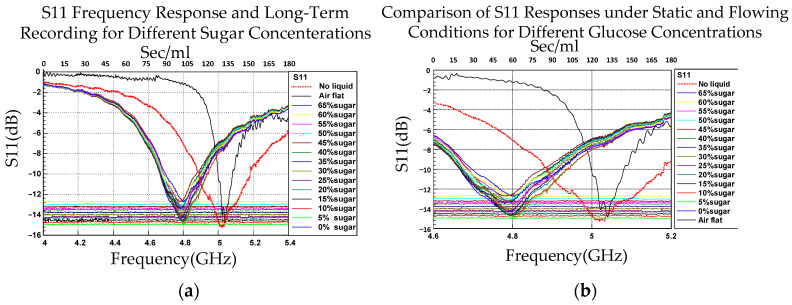
(**a**) Steady-state mean values (0–180 s). (**b**) Enlarged view of (**a**).

The blood flow simulation platform reliably acquired flow-related indicators—the drop rate, flow rate, and pressure—within a fixed measurement window. These indicators were used to characterize flow behavior at various glucose concentrations and served as the basis for simulating human blood flow. For each concentration, the initial transient segment of the signal was excluded, and the average value across the steady-state region was considered a representative indicator (steady-state mean). Figure 26 presents the steady-state mean values of the *S*_11_ frequency response during the 180 s measurement period. Because the steady-state mean is a time-domain statistical quantity, the steady-state mean values of the flow measurements are presented as horizontal reference lines in Figure 26a. These lines enable correlation of the time-domain flow measurements with the reduction in frequency-domain CSRR resonance during the frequency sweep measurements. Figure 26b presents an enlarged view of Figure 26a, depicting the curves in the −15.5 to −12.7 dB interval.

Table 7 summarizes the descriptive statistics of *S*_11_ values measured under continuous-flow conditions at Brix concentrations ranging from 5% to 65%. The mean, SD, median, coefficient of variation, and coefficient of skewness were calculated to characterize the concentration-dependent response, measurement dispersion, and data distribution. The results revealed that the mean *S*_11_ value was more negative when the Brix concentration was higher. The relatively small SD values indicated favorable measurement stability and repeatability, whereas the small skewness values suggested that the distribution of data approached a symmetric distribution. All values are calculated as the average of five repeated measurements for each concentration under controlled experimental conditions.

**Figure 27:**
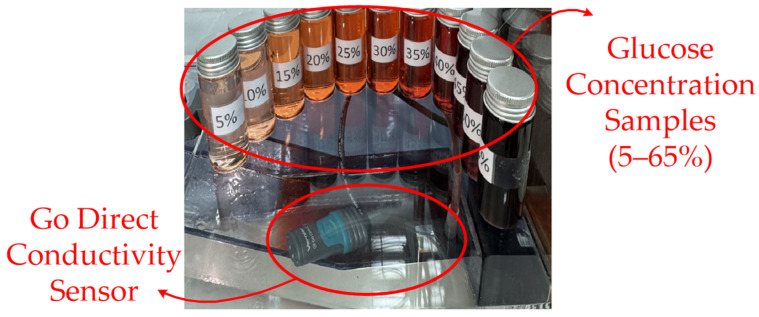
Glucose concentration samples (5–65%) and conductivity sensor.

Hysteresis flow unit: The hysteresis flow unit was incorporated as an auxiliary module to measure the electrical conductivity of the aqueous solution being tested, thereby enabling the estimation of concentration-dependent variation in ionic content. In practice, the unit provides a reference indicator of electrolyte-related changes in the test liquid. This indicator can be used to interpret concentration-dependent flow behavior and determine whether the observed responses arise from ionic effects or other mechanisms (e.g., viscosity-driven hydraulic resistance). Figure 27 depicts conductivity measurement setup with glucose concentration samples ranging from 5% to 100%. The conductivity sensor is used to measure variations in electrical conductivity corresponding to different concentrations.

## 5. Conclusions, Implications, Limitations, and Potential Improvements

In this study, various glucose concentrations were estimated by measuring the *S*_11_ values of the proposed system’s CSRR and analyzing the obtained concentration–resonant frequency and concentration–resonance magnitude plots. The experimental results demonstrated the feasibility and potential of this approach. The averaged statistical results show that the mean and median values are highly consistent, with a low standard deviation and coefficient of variation, indicating stable and reliable measurement performance (Mean = −13.7687 dB, Median = −13.7704 dB, SD = 0.028, CV = −0.0280 dB, Skewness = 0.166) The skewness value suggests an approximately symmetric distribution of the measured S11 responses. Conductivity may be used to examine potential hysteresis effects when the concentration of a solution is swept upward and downward under fixed flow conditions. The proposed CSRR-based system is passive and non-Faradaic and therefore does not generate an electrochemical conductivity effect in the sample being investigated. The CSRR interrogates the sample through electromagnetic coupling rather than by injecting current to induce an electrochemical reaction. Thus, CSRR-based measurements neither alter the ionic state of the liquid sample nor induce electrical hazards in biological applications.

The measurement workflow described in this article can be extended to saline solutions, enabling evaluation of how electrolyte concentration influences resonant features. The platform can also be applied to oils with different concentrations, grades, or compositions (e.g., contaminated water and industrial oils with different degrees of aging), for which viscosity and dielectric properties vary substantially and may provide complementary signatures in the CSRR response and flow-resistance metrics. Comparative measurements of different liquid types (aqueous electrolytes, viscous oils, and mixed or contaminated liquids) may help establish the generality of the proposed approach and clarify the roles of the dielectric permittivity, dielectric loss, and viscosity in determining the sensing results. Future research may optimize the measurement procedure and increase sample size to enhance measurement precision and accuracy.

A limitation of this study is that the comparative analysis did not systematically cover broader benchmarking dimensions, such as operating frequency, sensing mechanism, sensing range, sample type, and implementation complexity. This omission is mainly due to the substantial heterogeneity among prior studies in terms of design objectives, test conditions, and application scenarios; establishing a unified comparison would require standardized and application-relevant evaluation criteria. In addition, several key descriptors for metrological validation remain to be quantified through further experiments, including the limit of detection, effective resolution under instrumental noise, uncertainty propagation, temporal drift, inter-device reproducibility, and environmental robustness. Therefore, the claims of the present work are limited to the response characteristics demonstrated under controlled test conditions. Future work will focus on developing a comprehensive benchmarking framework and performing standardized evaluations to quantify these metrics under comparable and application-relevant conditions.

## 6. Patents

The work reported in this manuscript has resulted in the following patent: Wu, W.L.; Peng, K.-C. Inspection Apparatus for Blood Flow Simulation in Blood Vessels. R.O.C. Invention Patent I917277, 1 March 2026.

## Figures and Tables

**Table 1:** Methods for measuring the dielectric constant.

Method	Example Applications	Advantages	Disadvantages
Transmission line method [6]	Circuit implementations with wide bandwidth	Broadband measurement; applicable within actual circuits	High equipment and calibration requirements
Reflection method [7]	High-frequency and millimeter-wave bands	Simple measurement procedure; suitable for high frequencies	Limited to surface measurements; stringent calibration required
Free space method [8]	High-frequency to millimeter-wave bands	Noncontact measurement; suitable for high-frequency and millimeter-wave applications	Strict environmental requirements; expensive equipment
Resonance method [9]	High-precision applications; high-Q materials	High measurement accuracy	Limited measurement range; expensive equipment
Coplanar Waveguide method [10]	Microwave and millimeter-wave bands	Suitable for high-frequency measurement; high integration capability	Requires specific waveguide structures; complex design

**Table 2:** Comparison of $S_{11}$ and $S_{21}$ measurements.

Characteristic	$S_{11}$ (Reflection Parameter)	$S_{21}$ (Transmission Parameter)
Measurement focus	Reflects characteristics at the input port; impedance matching	Reflects characteristics of transmission from the input port to the output port; transmission efficiency
Resonance characteristic	Lowest reflection	Highest transmission efficiency
Concentration– resonant frequency relationship	Increases with increasing concentration	Increases with increasing concentration
Concentration–resonance magnitude relationship	Exhibits a quadratic trend	Resonance magnitude decreases with increasing concentration
Sensitivity	Sensitive to variation in impedance matching	Sensitive to signal transmission loss

**Table 3:** Calculated tendency and statistical dispersion of datasets used in Figure 13a, Figure 14a and Figure 16a.

Glucose Solutions %	Resonant Frequency from Figure 13/(GHz)	S11 Magnitude from Figure 14/(dB)	Resonant Frequency from Figure 16/(GHz)
5%	4.766212	−13.22772	4.714475
10%	4.769967	−13.2244544	4.722601
15%	4.773721	−13.242532	4.734964
20%	4.780005	−13.278355	4.747501
25%	4.783802	−13.335063	4.757171
30%	4.791281	−13.378155	4.776093
35%	4.793704	−13.357848	4.814476
40%	4.795212	−13.358715	4.833208
45%	4.798713	−13.35063	4.849914
50%	4.798865	−13.3075063	4.860075
55%	4.806373	−13.262331	4.875171
60%	4.809325	−13.2445623	4.889736
65%	4.812501	−13.1596582	4.890226
Mean	4.790744692	−13.2867330	4.805046877
SD (Secure Digital)	0.014433288	0.06406354	0.06301544
μ + 3σ	4.834044558	−13.0945424	4.994093197
μ + 2σ	4.819611269	−13.1586060	4.931077757
μ + 1σ	4.805177981	−13.2226695	4.868062317
μ − 1σ	4.776311404	−13.3507966	4.742031437
μ − 2σ	4.761878115	−13.4148601	4.679015997
μ − 3σ	4.747444827	−13.4789237	4.616000556

**Table 4:** Comparison of state-of-the-art microwave glucose concentration sensors based on S-parameter variations.

Study	Zhu et al., Sensors 2024 [11]	Mansour et al., Sensors 2025 [12]	Hassain et al., Sensors 2025 [13]	Present Study
CSRR configuration/type	Electrically small patch antenna with dual CSRRs integrated into the ground plane	Planar 16-cell double-honeycomb–CSRR array on FR-4 and Rogers substrates	Modified inductive-stub-coupled CSRR with an added inductive stub	Patch-type CSRR microwave resonant sensor with an integrated simulated vascular flow platform
Sensing target	NaCl concentration	Blood glucose concentration	Blood glucose concentration	Glucose solution concentration under static and dynamic conditions
Measurement mode	Direct immersion of the sensor in the liquid without an additional container	Noninvasive in vivo measurement with single- and dual-finger contact and in vitro validation	Noninvasive simulation with a multilayer finger model	Static and dynamic measurements, with auxiliary parameters including pressure, and electrical conductivity
Operating frequency	~1.37 GHz	1.5–3 GHz; amplitude operating point on Rogers substrate: ~1.51 GHz	~2.3 GHz (dual-resonance behavior)	4.8–5.0 GHz; designed resonance: ~5 GHz
Primary sensing parameters	*S*_11_ response and resonant frequency	*S*_21_ amplitude response and frequency shift	Frequency shift and *S*_21_ amplitude variation	*S*_11_ as the primary parameter, with additional analysis of *S*_21_ resonant characteristics
Key results and sensitivity	The sensor was capable of measuring NaCl concentrations from 5‰ to 35‰, with a maximum sensitivity of 0.367 kHz/(mg/L) and an electrical size of approximately 0.23λ	The frequency sensitivity of the FR-4-based sensor was 2.005 MHz/(mg/dL), whereas the amplitude sensitivity of the Rogers-based sensor reached 9.35 × 10^−2^ dB/(mg/dL). Validation in 31 participants yielded an *R*^2^ value of 0.980, a root mean square error of 2.316 mg/dL, and an accuracy of 97.833%.	At the optimal stub gap of 2 mm, the frequency sensitivity was 0.086 MHz/(mg/dL) and the amplitude sensitivity was 0.02 dB/(mg/dL); compared with the conventional CSRR, these values represented improvements of approximately 168% and 72%, respectively.	Over a glucose concentration range of 5–65%, changes in glucose concentration could be identified from the resonant frequency shift, whereas the integrated flow platform enabled evaluation of the drop rate, pressure, and flow behavior.
Key advantages and distinguishing features	Its advantages include a small electrical size, a simple structure, and direct immersion-based measurement. However, the sensing target is saline solution rather than glucose, and the application is limited to solution concentration measurement; noninvasive biological sensing is not possible.	Its main advantages are the demonstration of noninvasive human validation and the comparison of the substrate’s effects on frequency and amplitude sensitivity. However, the sensor’s structure is relatively complex, and the study focused on dual-finger contact-based biomedical monitoring, which differs from the sensing orientation of the present study’s liquid platform–based approach.	Its main advantage lies in its use of an inductive stub to enhance field confinement and coupling efficiency, providing valuable insight into how a conventional CSRR can be improved. However, the study focused primarily on simulation-based evaluation.	The proposed system is distinguished by its ability to function under both static and dynamic conditions and its integration of flow behavior together with electromagnetic sensing. The system approaches a prototype for needle-free, long-term glucose monitoring by combining liquid electromagnetic sensing with a flow platform.

**Table 5:** Description and specifications of the adopted measurement devices.

System Module	Device	Primary Function	Key Specifications
Hysteresis flow unit	Flow rate was determined using a drop counter (Go Direct Drop Counter, Vernier Software & Technology, Beaverton, OR, USA).	Measures the conductivity of aqueous solutions to estimate variation in ionic concentration	Conductivity range: 0–20,000 µS/cm; resolution: 0.01 µS/cm; accuracy (factory-calibrated): ±1%; temperature compensation: 5–35 °C; operating temperature: 0–80 °C; response time: 98% of the final value is reached within 5 s
Pressure	Pressure measurements were performed using a gas pressure sensor (Go Direct Gas Pressure Sensor, Vernier Software & Technology, Beaverton, OR, USA)	Measures pressure (serving as both a driving force and flow resistance indicator); enables monitoring of mean pressure and pressure fluctuations	Measurement range: 0–210 kPa; maximum pressure: 405 kPa; accuracy: ±4 kPa (factory-calibrated); response time: 10 ms; internal volume: 0.8 mL
Drop count sensor	Electrical conductivity was measured using a conductivity sensor (Go Direct Conductivity Sensor, Vernier Software & Technology, Beaverton, OR, USA).	Records drop counts and converts them into volumes; compatible with pH, conductivity, and oxidation–reduction potential measurements for titration or synchronized acquisition	Maximum counting rate: 6 drops/s. Volume Based on Drop Count

**Table 6:** Drop rate and time per drop for glucose solution concentrations from 5% to 65% (each drop had volume of 1 mL).

Concentration 8 mm	1 s/A Drop (Drop/s)	Seconds Per Drop (s/Drop)	Total Delivered Volume in 180 s (mL)
5%	6.5	0.153	1170
10%	6.102	0.166	1098.36
15%	5.023	0.181	904.14
20%	5.045	0.2	908.1
25%	4.501	0.223	810.18
30%	4.065	0.250	731.7
35%	3.512	0.285	632.16
40%	3.043	0.334	547.74
45%	2.501	0.4	450.18
50%	2.098	0.5	377.64
55%	1.532	0.666	275.76
60%	1.078	0.1	194.04
65%	0.565	2	101.7

**Table 7:** Descriptive statistics of *S*_11_ values measured under continuous-flow conditions at various glucose concentrations.

Glucose%	Mean	SD	Median	Coefficient of Variation (CV) (%)	Skewness
5%	−14.93779	0.03158	−14.9399	−0.002114	0.20009
10%	−14.70746	0.04501	−14.714	−0.00306	0.43564
15%	−14.50444	0.02229	−14.5049	−0.001536	0.06162
20%	−14.29943	0.02293	−14.29849	−0.001603	−0.1236
25%	−14.19122	0.0289	−14.1925	−0.002036	0.1319
30%	−13.99103	0.0317	−13.9925	−0.002266	0.13866
35%	−13.77247	0.02394	−13.7747	−0.001738	0.2785
40%	−13.50458	0.02401	−13.5102	−0.001778	0.70109
45%	−13.34246	0.02763	−13.3449	−0.00207	0.2642
50%	−13.2165	0.02263	−13.2159	−0.001712	−0.08019
55%	−12.97934	0.02965	−12.9785	−0.002284	−0.0859
60%	−12.83169	0.02829	−12.8349	−0.002204	0.3398
65%	−12.71486	0.02507	−12.7139	−0.001972	−0.11554

## Data Availability

The original contributions presented in this study are included in the article. Further inquiries can be directed to the corresponding author.
